# Natural radioactivity levels of some medicinal plants commonly used in Ghana

**DOI:** 10.1186/2193-1801-2-157

**Published:** 2013-04-11

**Authors:** Lordford Tettey-Larbi, Emmanuel Ofori Darko, Cyril Schandorf, Alfred Ampomah Appiah

**Affiliations:** 1 Graduate School of Nuclear and Allied Sciences, University of Ghana, P. O. Box AE 1, Atomic Energy, Kwabenya, Accra, Ghana; 2 Radiation Protection Institute, Ghana Atomic Energy Commission, Box LG 80, Legon, Accra, Ghana; 3 Centre for Scientific Research into Plant Medicine, P. O. Box 73, Mampong-Akuapem, Ghana

**Keywords:** Medicinal plants, Natural radioactivity, Gamma-ray spectrometry, Annual committed effective dose

## Abstract

Natural radioactivity levels in some selected medicinal plants commonly used in Ghana from the Centre for Scientific Research into Plant Medicine were investigated to determine the activity concentration and the annual committed effective dose due to naturally occurring radionuclides of ^238^U, ^232^Th and ^40^K. The activity concentration was determined using gamma-ray spectrometry. The results of the analysis indicated an average activity concentration of ^238^U, ^232^Th and ^40^K in the medicinal plants to be 31.8±2.8 Bq kg^-1^, 56.2±2.3 Bq kg^-1^ and 839.8±11.9 Bq kg^-1^ respectively. *Khaya ivorensis* recorded the highest activity concentration of ^238^U and ^232^Th while *Lippia multiflora* recorded the highest activity concentrations of ^40^K. The total annual committed effective doses ranged from 0.026±0.001 to 0.042±0.002 mSv a^-1^ with an average value of 0.035±0.001 mSv a^-1^. The average annual committed effective dose due to ingestion of the natural radionuclides in the medicinal plant samples were far below the world average annual committed effective dose of 0.3 mSv a^-1^ for ingestion of natural radionuclides provided in UNSCEAR 2000 report. Therefore, the radiological hazard associated with intake of the natural radionuclides in the medicinal plants is insignificant. The results provide baseline values which may be useful in establishing rules and regulations relating to radiation protection as well as developing standards and guidelines for the use of medicinal or herbal plants to the appropriate authorities.

## Introduction

Naturally occurring radioactive materials (NORMS) are found in every constituent of the environment; air, water, soil, food and in humans. According to the International Food Safety Authorities Network (INFOSAN 2011), plants used as food commonly have ^40^K, ^232^Th and ^238^U and their progenies. It is expected that likewise would be found in plants used for medicinal purposes since plants are the primary pathway of natural radionuclides entering into the human body through the food chain. Radionuclides and their decay products from ^238^U and ^232^Th series together with ^40^K are terrestrial primordial radionuclides, which originated from the earth’s crust and are the sources of natural radioactivity in the environment (Kessaratikoon and Awaekechi 2008).

All over the world, raw parts of plants and their extracts are used in the medicinal products. It is also estimated that 25% of modern medicines are derived from medicinal plants of which most of them are flowering plants (Gurib-Fakim and Kasilo 2010). There are over 250,000 flowering plant species that serve as resources for the production of new drugs, orthodox or traditional. The World Health Organisation (WHO) define traditional medicine as comprising therapeutic practices that have been in existence, often for hundreds of years, before the development and spread of modern medicine and are still in use today (WHO 1991).

A survey by the WHO indicates that about 70 – 80% of the world population rely on traditional medicine mainly from plant sources due to the increasing emphasis on advocacy on Primary Health Care or Basic Health Care (Brown 1992); (WHO 1998). In Ghana, 70% of the populations rely on herbal medicines where the Practitioner to Patient ratio is 1:400 in case of traditional medicine as against 1:12000 in modern medicine even though the lack of scientific proof of the effects of traditional medicine has often made scientists doubt their efficacy (Serfor-Armah et al. 2003); (Haq 2004); (Owusu-Afriyie 2005). However, plant based medicines present a high efficacy, safety and lesser side effects and offer remedy for age-related disorders like memory loss, osteoporosis, immune disorders for which no modern medicine is available (Kamboj 2000).

The therapeutic effect of these medicinal plants for the treatment of various diseases is based on the organic constituent (such as essential oil, vitamins, glycosides, etc.) present in them (Desideri et al. 2009), although, certain inorganic elements (example Al, Br, Ca, Cl, Mn, Mg, etc.) have been considered as essential in the formation of active constituent which are responsible for the curative properties of the medicinal plants (Serfor-Armah et al. 2001). These stable inorganic elements like the unstable ones (radioactive element) find their way into these plants during the process of photosynthesis. Natural radionuclides are transferred and cycles through natural processes and between the various environmental compartment by entering into the ecosystem and food chain through direct or indirect contamination of natural radionuclides (Adewumi 2011). This processes involving photosynthesis include the absorption of soluble radionuclides in soil-water by root uptake, direct deposition from the atmosphere and re-suspended natural radionuclide from the soil. The main factors controlling the rate of plant root uptake of natural radionuclides are the uptake potential for different natural radionuclides and the availability of natural radionuclides in the soil. The rate of natural radionuclides uptake is highly dependent on the activity concentration in the soil. Also, the root uptake depends on soil properties such as pH, mineralogical composition, organic matter content and nutrient status as well as metabolic and physiological characteristic of the plant species (IAEA 2006). Obviously, plants uptake of radionuclides is one of many vectors for the migration of natural radionuclides into humans from the environment via the food chain.

The study of the radioactivity levels of plants in the environment are of interest within ecological and plant evolution under certain conditions of geochemical point of view and adaptation and it thus provide information in the monitoring of environmental radioactivity (Mukhammedov and Tillaeva 2005). The study of NORMS in plants is also not only important because of the risk associated with it but also from the fact that some of them can be used as biochemical tracer in the human food chain.

The role of NORMS in animal and plant metabolism has long been established and available in literature, but the effect and influence of these NORMS on administration of medicinal plants had received relatively little attention without due regard to possible side effects possibly because medicinal plants or herbal plants are not considered in the group of edible plants that have been studied in the past by nutritionist, although many edible plants used as spices or fruits such as ginger, onion, pawpaw and mango etc., have medicinal properties and the ingestion of NORMS through the use of medicinal plants had not been recognized or considered significant in terms of quantity. These factors have therefore contributed little or no information on the levels of the natural radioactive materials constituent of these medicinal or herbal plants preparation and their extracts in Ghana.

Epidemiological studies have not demonstrated adverse health effects in individuals exposed to small doses (< 0.1 Sv) delivered in a period of many years with the exception of radiogenic health effects (primarily cancer) which is evident in epidemiological studies for only doses exceeding 0.05-0.1 Sv delivered at high dose rates (HPS 2004). However the linear non-threshold (LNT) model indicates levels of risk to all levels of radiation (ICRP 1991,2005). Although, some scientists believe that since humans evolved and survived through this evolutionalised environment which consists of some levels of radiation, some low levels of radiation are beneficial to the human (Hormesis) (Cuttler 2004).

Medicinal plants are administered in their raw form or in formulations such as solutions, tablets or capsules. Undoubtedly, the activity concentrations of NORMS in herbal formulations are quite lower than in the raw plants due to the preparation processes which inevitably remove some of the radionuclides. The health effects of radiation exposures to NORMS from intake of medicinal plants and herbal preparations in relation to the levels of NORMS in medicinal plants may be associated with most forms of leukemia and with cancer of many organs such as the bone, lung, breast and thyroid in the long term, but not with all organs including prostate. Example; approximately 10-15% of ^210^Pb and ^214^Pb ions, 99% of ^226^Ra and ^228^Ra, ^214^Bi (bone seeker) and ^210^Po (soluble) reaches the blood and/or the lung fluid stream and are distributed to the whole body and exchanged with calcium in the mineral of skeletal tissues thereby making blood, bone and lung critical organs (UNSCEAR 1988,2000); (Cember and Johnson 2009). Also, ^238^U has affinity for electron donor (e.g. oxygen) and therefore deposits itself in the tissues of the lung and lining on the bones marrows which can lead to cancer of the blood (leukemia). ^232^Th which is also an alpha particle emitter settles in the lining of the bones which can lead to bone cancer (Cember and Johnson 2009). Although ^40^K is not considered to be of radiological significance, the body’s control system of blood pressure and volume is dependent on the activity concentration of potassium in the body and an abnormality in the control of this activity concentration level in the body by the body system presents a general potential for High Blood Pressure and subsequent cancer induction with a lifetime cancer Mortality Risk of 0 814Bq^-1^ for ingestion (ANL 2005). However, studies have shown that the total amount of potassium in the human body is maintained constant at a fixed body mass. The activity concentration of K-40 in potassium in plants extracts and raws is not different from those in foods or in the human body. Intake of additional potassium through food intake simply results in a reduction in the biological half-life of potassium in the body and resultantly no increase in the dose from K-40. This implies that potassium is homeostatic within the body (Tahir and Alaamer 2008); (Tahir et al. 2010).

The objectives of this study are to determine the specific activity concentrations and the annual effective doses due to ingestion of NORM due to ^238^U, ^232^Th and ^40^K present in some selected medicinal plants commonly used in Ghana from the Centre for Scientific Research into Plant Medicine (CSRPM) at Mampong-Akuapem in the Eastern Region and to assess the radiological risk associated with the use of these medicinal plants.

## Materials and methods

### The sampling area

The choice of the sampling site was an important factor in this study. Prominence was placed on the choice of a clinic that has the highest production and use of medicinal plants in Ghana. The Centre for Scientific Research into Plant Medicine (CSRPM) was established in 1975 by the Government of Ghana through the pioneering work of Dr. Oku Ampofo, a Ghanaian allopathic medical practitioner. Today, it is a leading research institution in Africa and its core business is into Research and Development of herbal medicines in Ghana and Africa as a whole (CSRPM 2011).

The Centre is located at 5° 55' 04.58"N and 0° 08'02.89"W at an elevation of 1,488 ft and an eye altitude of 1,575 ft (Google Earth 2012) in the mountainous forest savanna vegetation area of Mampong, Eastern Region of Ghana. The centre is off Mampong-Akropong road, which lies within the wet semi-equatorial zone, characterized by double maxima rainfall in June and October with relatively low temperatures and high humidity through the year.

The Centre offers the following services: clinical laboratory services, production and dispensing of herbal medicines to patients, raising of medicinal plant seedlings for sale to out growers, formulation and production of herbal extracts for industries, safety, efficacy and quality assessment of herbal products from manufacturers and herbal practitioners and provision of library and internet café for research into plant medicine.

### Sample collection

Three samples (1 kg each) of Eight (8) different medicinal plants parts samples (Table 1) used at the Centre were collected between the month of October and December, marking the end of the raining season and the beginning of the dry season which allows for fresh sampling and a good weather condition for drying the samples. The medical use of these plant parts is also shown in Table 1. The samples were transferred in labeled polyethylene bags from the Centre to the laboratory.

**Table 1:** **Physical data of plant samples used**

Name of plant sample	Local name (Akan)	Part of plant sampled	Medical use
*Lippia multiflora*	Saresonunum	Leaves	Hypertension, lactation failure, insomia, conjunctivitis,
*Croton membranaceus*	None	Roots	Prostatic hypertrophy, measles
*Cassia sieberiana*	Prongkese	Root-bark	Menstrual pain, abdominal pain, (Pain killer)
	Poto rodom		
*Bridelia ferruginea*	Opan fufuo	Leaves	Hypertension, diabetes
*Mondia whitei*	Mondi	Roots	Improve sex hormones (low sperm count), STD, Stroke
*Blighia sapida*	Takwa dua	Stem-bark	Migraine, diarrhoea
	Ankye fitaa		
*Alstonia boonei*	Nyame dua	Stem-bark	Malaria, rheumatism, boil
*Khaya ivorensis*	Odupon	Stem-bark	Fever, anemia

### Sample preparation

The samples were open air dried on trays for a period of one week and then oven dried at a temperature of 105°C (± 5°C) for 2 to 4 hours at the laboratory. The oven dried samples were then grounded into fine powder with a stainless steel ball grinder. The prepared samples, in powdered form, were packed into weighed one (1) liter Marinelli plastic beaker, hermetically sealed, reweighed and stored prior to counting (Scheibel and Appoloni 2007); (Changizi et al. 2010); (Olatunde et al. 2011). The containers were sealed to avoid any possibility of out-gassing of radon and kept for a period of 1 month to make sure the samples attained radioactive equilibrium between Ra-226 and its decay products in the uranium series, and Ra-228 and its decay products in the thorium series (Tahir and Alaamer 2009).

### Analysis of the medicinal plant samples

The samples were counted using a gamma-ray spectrometry. The gamma-ray spectrometry system consists of an N-type high purity germanium (HPGe) detector Model GR 2518 (Canberra Industries Incorporated) with a relative efficiency of Twenty-five percent (25%) to NaI detector, 1.8 keV energy resolution (FWHM) at energy peak of 1333 keV of ^60^Co isotope, a peak-to-Compton ration of 55:1. The bias voltage of -3000 V was supplied by a HV Power Supply (Model 13103) through an Uninterrupted Power Supply (UPS). The detector was cooled in liquid nitrogen at -196°C (77K) provided in a 25 liter Dewar with ambient temperature around the detector being between 16°C and 27°C. The detector was connected to a Spectroscopy Amplifier (model 2020, Canberra Industries Incorporated) and a computer based PCA-MR 8192 ACCUSPEC Multi-Channel Analyzer (MCA) and a Microsoft soft window software ORTEC MAESTRO-32 for spectrum acquisition, evaluation and analysis. The detector was mounted in a cylindrical lead shield with internal diameter of 24 cm, thickness of 5 cm and a height of 60 cm. The lead shield consist of a 0.3 cm thick each of various layers of copper, cadmium and plexiglass.

The energy and efficiency calibration of the system was carried out before sample analysis using the multinuclide reference standard solution supplied by the International Atomic Energy Agency, IAEA. This was to enable identification and quantification of the radionuclides. The standard and the sample were counted for a period of 36,000 seconds to acquire spectral data for a better counting statistics and evaluation. The activity concentration of ^238^U, ^232^Th and ^40^K were determined after correction for background and inhomogeneities (Oresengun et al. 1993); (Gilmore and Hemingway 1995).

The specific activity concentration of ^238^U, ^232^Th and ^40^K in the medicinal plants were determined from the quantitative analysis of the spectra acquired from the Gamma-ray spectrometry using the Gamma-ray spectrum analysis software, Ortec MAESTRO–32 at specific energies. ^238^U was calculated from the average of ^214^Pb at energies of 251.9 keV and 295.2 keV and ^214^Bi at energies of 609.3 keV and 1764.5 keV. ^232^Th was determined from the average of ^208^Tl at energies of 2614.5 keV and 583.2 keV, ^212^Pb at the energy of 238.6 keV and ^228^Ac at the energy of 911.2 keV and ^40^K at 1460.0 keV.

The specific activity (*A*_*sp*_*(E,i)* in Bq kg^-1^) of the radionuclide *i* in the samples were calculated after decay correction using the expression in equation (1) (Ebaid 2010).1

where; *N*_*sam*_ (E,_i_) is the net counts for the radionuclide *i* at energy *E, ε*_*γ*_*(E)* is the photopeak efficiency at energy *E, T*_*c*_ is the counting live-time (s), Pγ(E, i) is the gamma emission probability of the radionuclide *i* for a transition at energy *E*, *M*_*sam*_ is the dry-weight of samples (kg).

Having obtained the values for the specific activity concentrations of the individual naturally occurring radionuclides in the medicinal plants, the average annual committed effective dose, *E*_*ave*,_ for ingestion of NORMS in the medicinal plants were calculated using the expression in equation (2).2

Where *DCF*_*ing*_ is the dose convection factor for ingestion, for each radionuclide (i.e., 4.5 × 10^-5^ mSv Bq^-1^, 2.3 × 10^-4^ mSv Bq^-1^ and 6.2 × 10^-6^ mSv Bq^-1^ for ^238^U, ^232^Th and ^40^K respectively for an adult (UNSCEAR 2000), *I*_*p*_ is the consumption rate from intake of NORMS in medicinal plants and *A*_*sp*_ is the activity concentration in the plant sample.

Generally in Ghana, the average percentage plant material in grams used in herbal preparation or products is five percent (5%) of which the average dosage is two (2) table spoons full (30 ml), three (3) times daily (Personal communication, Appiah A. A. 2012). This means that, for every 100 ml of herbal product, 5 g of plant material is used and also since one (1) table spoon full is equivalent to 15 ml, two (2) table spoons full, three (3) times daily is equivalent to 90 ml. Therefore, on the basis of this information and the non-availability of a well-accepted consumption rate for medicinal plants, a consumption rate of 1.8 kg yr^-1^ was assumed for all the medicinal plants used in this study, assuming that a patient needs 100 ml day^-1^ (an upper average dosage) of the herbal preparation or product during the treatment period.

## Results and discussions

### Activity concentrations in the medicinal plants

**Figure 1:**
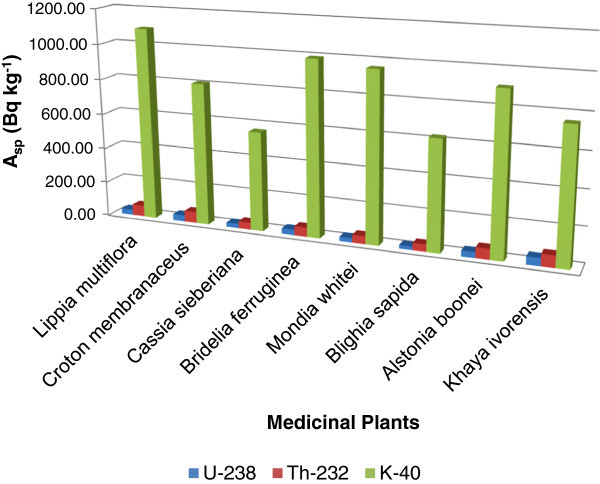
**Comparison of the activity concentration (A**_**sp**_**) of**^**238**^**U,**^**232**^**Th, and**^**40**^**K in the various species of the medicinal plant samples.**

The activity concentration of ^238^U in the medicinal plants ranges from 20.42±1.99 to 46.89±2.13 Bq kg^-1^ with an average value of 31.78±2.80 Bq kg^-1^. The highest activity concentration of ^238^U was recorded for *Khaya ivorensis* whilst *Blighia sapida* had the lowest activity concentration. For the activity concentration of ^232^Th, it varied from 42.02±1.71 to 70.60±3.37 Bq kg^-1^ with an average value of 56.16±2.32 Bq kg^-1^ in the medicinal plants. The highest and lowest activity concentration was recorded for *Khaya ivorensis* and *Blighia sapida* respectively. ^40^K recorded the highest activity concentration in all the medicinal plants compared to the activity concentration of ^238^U and ^232^Th observed. The activity concentration varied from 566.35±7.90 to 1093.11±17.02 Bq kg^-1^ with an average value of 839.80±11.86 Bq kg^-1^. *Lippia multiflora* recorded the highest activity concentration with the second highest being *Bridelia ferruginea* whilst the lowest was record in *Cassia sieberiana*. The activity concentrations of the various medicinal plants are presented in Table 2 and the activity concentration have been compared and represented in Figure 1.

**Table 2:** **Activity concentrations and average annual committed effective doses of**^**238**^**U,**^**232**^**Th and**^**40**^**K in the medicinal plant samples**

Sample	Activity concentration (Bq kg^-1^)			Average annual committed Effective dose (mSv a^-1^)
	^238^U	^232^Th	^40^K	
*Lippia multiflora*	32.2 ± 3.1	65.6 ± 3.1	1093.1 ± 13.1	0.014 ± 0.002
*Croton membranaceus*	36.1 ± 2.8	65.5 ± 2.4	808.8 ± 12.9	0.013 ± 0.002
*Cassia sieberiana*	25.8 ± 2.2	43.0 ± 1.7	566.4 ± 7.9	0.009 ± 0.001
*Bridelia ferruginea*	32.6 ± 2.6	53.3 ± 1.8	993.4 ± 12.6	0.012 ± 0.001
*Mondia whitei*	25.9 ± 3.0	46.9 ± 2.2	961.1 ± 11.2	0.011 ± 0.001
*Blighia sapida*	20.4 ± 2.0	42.0 ± 2.0	625.5 ± 10.4	0.009 ± 0.001
*Alstonia boonei*	34.4 ± 4.6	62.4 ± 3.4	911.1 ± 17.0	0.013 ± 0.001
*Khaya ivorensis*	46.9 ± 2.1	70.6 ± 1.9	759.1 ± 9.9	0.014 ± 0.002

The variation of the activity concentration of ^238^U, ^232^Th, and ^40^K in the different medicinal plant samples may be due to the fact that, the activity concentrations of ^238^U, ^232^Th, and ^40^K differ geographically from one soil of cultivation to another and some plants also absorbs certain elements more than others. Observably, the activity concentration of these radionuclides also varies with their half-life. Thus, the longer the half-life, the higher is the activity concentration of the radionuclides. This means that, the ratio of ^232^Th to ^238^U in these medicinal plant samples would to be greater than one as reported for other environmental samples (Miah et al. 1998). However, the activity concentration of ^40^K is usually high compared to ^238^U and ^232^Th and forms an integral part of all organic constituents. ^40^K in the samples varied of the order of about 10^1^ to 10^3^ Bq kg^-1^ more than ^238^U and ^232^Th.

The high activity concentration of Potassium recorded for *Lippia multiflora* and *Bridelia ferruginea* can significantly aid in its therapeutic purposes for the treatment of High Blood Pressure since patients with High Blood Pressure have low concentration of Potassium in their blood stream (HBPI 2012). Although *Bridelia ferruginea* is used for the treatment of diabetes, almost all diabetic patients are hypertensive (Personal communication, Appiah A. A. 2012).

Published work in this field had involved other medicinal plants other than those considered for this study. However, comparing the results from this study with published data from some countries indicates that both the average activity concentration values obtained for ^238^U and ^232^Th in this study are higher than the other published work with the exception of ^40^K of which activity concentration from Brazil (Scheibel and Appoloni 2007) is higher than reported values in this study (Table 3).

**Table 3:** **Comparison of the activity concentration of**^**238**^**U,**^**232**^**Th and**^**40**^**K in the medicinal plants from this study with those from other countries**

Country	Activity concentration ( Bq kg^-1^)						Reference
	^238^U		^232^Th		^40^K		
	Range	Average	Range	Average	Range	Average	
**Ghana**	20.4–46.9	31.8	42.0–70.6	56.2	566.4–1093.1	839.8	This work
**Brazil**	-	-	<11.0–43.0	21.7	666.0–1216.0	976.3	(Scheibel and Appoloni 2007)
**Italy**	<0.1–7.3	0.4	-	-	5.4–3582.0	654.7	(Desideri et al. 2010)
**Nigeria**	14.7–16.2	15.6	7.0–11.4	8.5	66.8–70.2	67.9	(Olatunde et al. 2011)
**Serbia**	0.6–8.2	2.6	1.7– 15.1	7.4	126.0–1243.7	589.6	(Jevremovic et al. 2011)

The variations in the activity concentrations could be due to differences in the geological location of the plants and the radiochemical composition of the soils in which these medicinal plants are grown or cultivated since the levels of activity concentration of natural radionuclides are not normalized across the globe and the plants ability to absorb particular elements more than the others. The medicinal plants sampled for this study are located within the Mampong-Akuapem environs which is a mountainous forest area. It is therefore likely to exhibit high activity concentration of ^238^U and ^232^Th because of the presence of sedimentary and igneous rocks which are phosphate rich (IAEA 2003). The characteristics of the vegetation (forest area) of the area might have also contributed to the levels since the levels also depends on the soil organic matter content, soil to water ratio, site characteristics, rate and amount of rainfall, soil drainage and biochemical processes (Abu-Khadra and Eissa 2008). The high activity concentration of potassium present in the medicinal plants from Brazil (Scheibel and Appoloni 2007), the Mate tea, may be due to application of potassium containing fertilizers to the soil of cultivated area and/or the plant ability to absorb more potassium from the soil. High applications of potassium containing fertilizer are the cause of high activity concentration of ^40^K in most soil (Bhatti and Malik 1994). Since no potassium containing fertilizers were added to the soils of cultivation for *Lippia multiflora* and *Bridelia ferruginea*, the high activity concentration of potassium in these plants may be due to the plants ability to absorb potassium from the soil more than the other elements.

### Average annual committed effective dose in the medicinal plants

**Figure 2:**
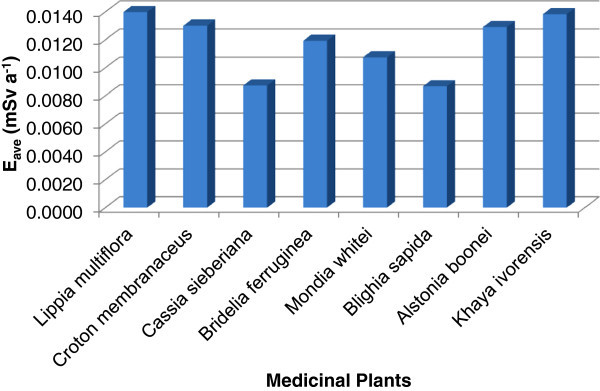
**The average annual committed effective dose (E**_**ave**_**) distribution in the various species of the medicinal plant samples.**

The average annual committed effective doses due to the ingestion of ^238^U, ^232^Th and ^40^K in the medicinal plant are also presented in Table 2. The average annual committed effective dose of ^238^U, ^232^Th and ^40^K varied from 0.009±0.001 to 0.014±0.002 mSv a^-1^ with an average of 0.012±0.001 mSv a^-1^. The highest average was recorded for *Lippia multiflora* whiles *Cassia sieberiana* and *Blighia sapida* have the lowest. Figure 2 shows the average annual committed effective dose distribution in the medicinal plant samples.

**Figure 3:**
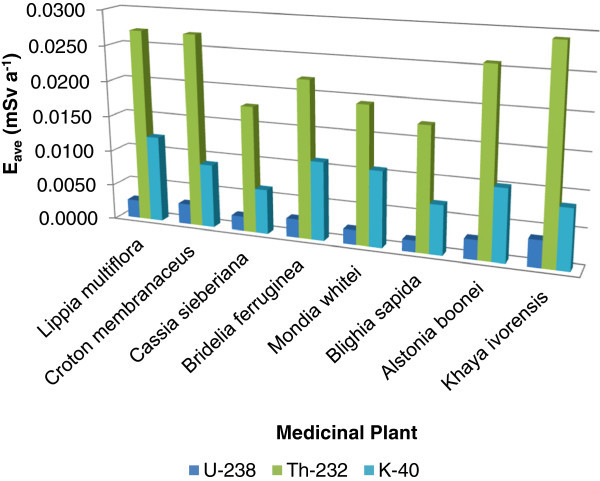
**Comparison of the annual committed effective doses (E**_**ave**_**) due to the natural radionuclides in the medicinal plant samples.**

**Figure 4:**
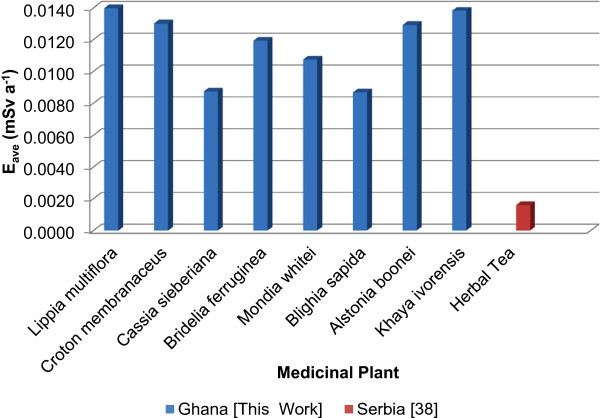
**Comparison of the average annual effective dose (E**_**ave**_**) due to NORMS in each medicinal plant sample with Herbal tea from Serbia.**

^232^Th contributes about 47% - 70% to the average annual committed effective dose more than ^40^K and ^238^U (Figure 3) although, the average annual committed effective dose due to ^238^U, ^232^Th and ^40^K for this study were relatively high compared to that which was reported for herbal tea in Serbia (Jevremovic et al. 2011) as shown in Figure 4. This is due to the differences in the assumed consumption rate value of medicinal plants applied to this study and that from Serbia. Other factors may be geographical location of the plants, the plants ability to efficiently absorb certain natural radionuclide more than others and whether the plant was in their raw state or product forms which have a direct effect on the activity concentration.

However, the calculated average annual effective dose to any individual in the population group due to the ingestion of natural radionuclides in the medicinal plants is far below the average radiation dose of 0.3 mSv a^-1^ received per head worldwide (UNSCEAR 2000).

## Conclusions

Natural radioactivity levels of some selected medicinal plants commonly used in Ghana from the Centre for Scientific Research into Plant medicine were investigated using gamma-ray spectrometry.

The average activity concentration obtained for ^238^U (31.78±2.80 Bq kg^-1^) and ^232^Th (56.16±2.32 Bq kg^-1^) in this study are higher compared to other published work with the exception of ^40^K (839.80±11.86 Bq kg^-1^) which recorded a lower activity concentration compared to that recorded from Brazil (Scheibel and Appoloni 2007). *Khaya ivorensis* recorded the highest activity concentration of ^238^U and ^232^Th whilst *Lippia multiflora* recorded the highest activity concentration of ^40^K. The high activity concentration of Potassium recorded in *Lippia multiflora* and *Bridelia ferruginea* can significantly aid in its therapeutic purposes in treating High Blood Pressure since patients with High Blood Pressure have low concentration of Potassium in their blood stream (HBPI 2012).

The corresponding average annual effective dose determined in this study to any individual’s organ or tissue in the population group due to the ingestion of natural radionuclides in the medicinal plants is far below the average radiation dose of 0.3 mSv a^-1^ received per head worldwide due to the ingestion of natural radionuclide (UNSCEAR 200). The result presents insignificant annual committed effective dose due to the use of these medicinal plants in Ghana from the Centre for Scientific Research into Plant Medicine. Therefore the radiological hazard associated with intake of the natural radionuclides in the medicinal plants is insignificant. Hence, the medicinal plants samples from the Centre are considered safe in terms of the radiological hazard.
